# The Power of Malaria Vaccine Trials Using Controlled Human Malaria Infection

**DOI:** 10.1371/journal.pcbi.1005255

**Published:** 2017-01-12

**Authors:** Luc E. Coffeng, Cornelus C. Hermsen, Robert W. Sauerwein, Sake J. de Vlas

**Affiliations:** 1 Department of Public Health, Erasmus MC, University Medical Center Rotterdam, Rotterdam, The Netherlands; 2 Department of Medical Microbiology, Radboud University Medical Center, Nijmegen, The Netherlands; Imperial College London, UNITED KINGDOM

## Abstract

Controlled human malaria infection (CHMI) in healthy human volunteers is an important and powerful tool in clinical malaria vaccine development. However, power calculations are essential to obtain meaningful estimates of protective efficacy, while minimizing the risk of adverse events. To optimize power calculations for CHMI-based malaria vaccine trials, we developed a novel non-linear statistical model for parasite kinetics as measured by qPCR, using data from mosquito-based CHMI experiments in 57 individuals. We robustly account for important sources of variation between and within individuals using a Bayesian framework. Study power is most dependent on the number of individuals in each treatment arm; inter-individual variation in vaccine efficacy and the number of blood samples taken per day matter relatively little. Due to high inter-individual variation in the number of first-generation parasites, hepatic vaccine trials required significantly more study subjects than erythrocytic vaccine trials. We provide power calculations for hypothetical malaria vaccine trials of various designs and conclude that so far, power calculations have been overly optimistic. We further illustrate how upcoming techniques like needle-injected CHMI may reduce required sample sizes.

## Author Summary

Controlled human malaria infection (CHMI) in healthy human volunteers is an important and powerful tool in clinical malaria vaccine development. However, to obtain meaningful estimates of protective efficacy, it is important to include an appropriate minimum number of participants, while minimizing the risks and burden for volunteers. Existing power calculations have limited value due to important influential assumptions. To optimize power calculations for malaria vaccine trials, we developed a non-linear, Bayesian statistical model for parasite kinetics as measured by quantitative real-time polymerase chain reaction, using existing data from mosquito-based CHMI experiments. Using our model, we provide improved, robust power calculations for various hypothetical malaria vaccine trials, taking account of important sources of variation between and within individuals. We conclude that so far, power calculations for malaria vaccine trials have been overly optimistic. We further illustrate how upcoming techniques like needle-injected CHMI may reduce required sample sizes.

## Introduction

In 2015, malaria caused an estimated 438,000 deaths (236,000–635,000) [1], most of which were associated with *Plasmodium falciparum* (Pf) infections. Hypothetically, a malaria vaccine targeting the sporozoite parasite stage (pre-hepatic vaccine), hepatic parasite stage (hepatic vaccine), and/or blood stage parasites (erythrocytic vaccine) could prevent many such deaths. In the past ten years, over 40 malaria vaccines have reached the clinical trial stage. So far, only the RTS,S vaccine has shown promising results (30%–65% protection against clinical malaria) [2–6], with a recent large, multi-center phase three trial showing 45.7% protection against clinical malaria in infants and children aged 5–17 months over a period of 18 months after three vaccine doses [7]. In response, the World Health Organization has recommended RTS,S for pilot implementation studies in Africa [8]. However, before any malaria vaccine can be tested in the field, its efficacy and safety need to be evaluated in controlled settings, which is most often done by means of controlled human malaria infection (CHMI) [9,10].

CHMI is currently considered to be a powerful tool in clinical vaccine development, as it allows researcher to control the otherwise highly variable infection rates. It is traditionally conducted by exposing a limited number of volunteers to laboratory-reared *Anopheles* spp. mosquitoes carrying Pf sporozoites [10,11], and more recently, also through needle injection of a defined number of aseptic cryopreserved sporozoites [12,13]. Parasitaemia can be monitored by means of blood smear microscopy but increasingly by sensitive quantitative real-time polymerase chain reaction (qPCR) [14–17]. The traditional study endpoint is detection of blood stage parasites by microscopy, at which point a study subject is treated with a curative regimen of an anti-malarial drug [10]. Under these conditions and with some additional precautionary measures, CHMI studies are considered to be safe, though the risk of severe adverse effects and the burden of venipuncture up to three times per day have to be weighed against the benefits of the information to be gained [18–20]. Power calculations for CHMI-based vaccine trials are therefore critical to ensure an appropriate number of included participants to obtain meaningful estimates of protective efficacy.

Here we provide an optimized method for power calculations for CHMI-based hepatic and erythrocytic vaccine trials in malaria-naive individuals, using qPCR and bloodsmear data from mosquito-based CHMI experiments in 57 non-vaccinated individuals. We developed a novel non-linear model for parasite kinetics during the first two weeks of an experimental infection, based on an earlier statistical model for cyclical patterns in CHMI [21]. We implemented the model in a Bayesian hierarchical framework to capture important sources of variation both within and between individuals (i.e. by means of random effects), and extended the model to explicitly capture the processes leading to measurements below the qPCR detection limit (censoring) and termination of the experiment due to positive blood microscopy. With this model, we performed power calculations for hypothetical hepatic and erythrocytic malaria vaccine trials under varying assumptions about blood sampling schemes and vaccine impact on parasite growth.

## Results

**Fig 1:**
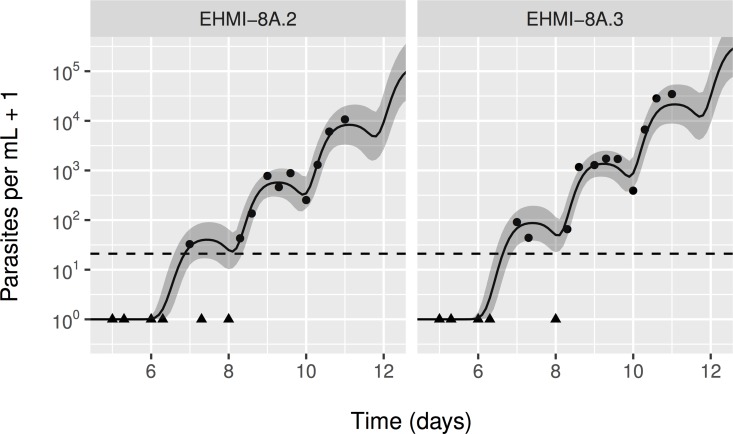
Example model predictions for blood parasite levels in a subset of individuals. Black bullets represent data points; black triangles are observations below the detection limit (dashed line). The solid black line represents the posterior mean. The shaded band around it represents the 2.5^th^ and 97.5^th^ percentiles of the predicted parasite concentrations, based on 8000 draws from the posterior distribution. Panel headers refer to unique identifiers for CHMI volunteers, which can also be found in the data (S1 File).

In Fig 1 we provide an example of the model fit to the data for a subset of individuals, and illustrates the cyclical pattern in both the data and model predictions. Note how censored observations (black triangles) coincide with model predictions near or under the qPCR detection limit (dashed line). Similar plots for all individuals can be found in S1 Fig. For a minority of subjects no clear cyclical pattern in parasitaemia levels was visible, resulting in relatively wide credible intervals for predicted parasitaemia levels. Data from one individual were excluded (ZONMW1.1270) because they were prohibitively divergent from the rest to effectively fit the model (first observation above detection limit at day ten and no clear cyclical pattern while this individual–like all others–had no vaccine protection). S2 Fig depicts the association between parasite concentration in the blood and probability of positive blood microscopy in comparison to the data, illustrating good agreement between the model and the data.

We estimated that the average number of first generation parasites among the 56 included study subjects was 635 Pf/ml (95%-BCI 406–945; Table 1). Inter-individual variation in the number of first generation parasites was estimated at a standard deviation (SD) of 1.40 on the natural logarithmic scale, which is equivalent to a relative standard deviation (RSD) of $\sqrt{\exp\left(1.40^{2}\right)-1}=247\%$. Assuming that infected hepatocytes release on average 6.0 merozoites per mL blood (based on 30,000 merozoites per hepatocyte and an average of 5 litres of blood per person, as assumed before [21]), we can deduce from the average concentration of first generation parasite that on average about 107 hepatocytes were infected and that each experimentally infected mosquito transmitted on average about 21 sporozoites (that successfully infected hepatocytes). The average time of appearance of first-generation parasites was at day 6.87 (95%-BCI 6.76–6.99), with little variation between individuals (SD 0.036, RSD 3.6%). The average multiplication rate of *P*. *falciparum* parasites within individuals was estimated at 11.8 per parasite cycle (95%-BCI 9.0–15.3), with inter-individual variation estimated at a SD of 0.47 on the natural logarithmic scale (RSD 50%).

**Table 1:** Parameter estimates for parasite kinetics in mosquito-based, controlled human malaria infection.

Parameter	Interpretation	Posterior mean	95%-BCI
Number of first generation parasites per mL blood			
*β*_1_	Population geometric mean	635	406–945
$\sigma_{\beta_{1}}$	Inter-individual variation (on natural logarithmic scale)	1.40	1.12–1.74
Parasite multiplication rate per cycle			
*β*_2_	Population geometric mean	11.8	9.0–15.3
$\sigma_{\beta_{2}}$	Inter-individual variation (on natural logarithmic scale)	0.47	0.27–0.70
Time of appearance of first generation parasites (days)			
*μ*_1_	Population geometric mean	6.87	6.76–6.99
$\sigma_{\mu_{1}}$	Inter-individual variation (on natural logarithmic scale)	0.036	0.025–0.046
*σ*_1_	Variation in time of appearance of individual first generation parasites (inversely related to rate at which parasite concentrations rise and fall within individuals)	0.24	0.20–0.28
Parasite generation time			
*μ*_2_	Blood stage duration (days)	1.19	1.04–1.34
*μ*_3_	Sequestration duration (days)	0.66	0.53–0.79
*μ*_23_	Total generation time (*μ*_2_ + *μ*_3_)	1.84	1.79–1.91
Measurement error			
*σ*_*y*_	Measurement error in natural logarithm of parasite blood concentration	0.98	0.90–1.07
Probability of positive blood microscopy			
*α*_TS_	Population-average log-odds of positive blood microscopy in case of 1 parasite per mL blood	-11.3	-15.1 –-8.4
*σ*_TS_	Inter-individual variation in log-odds of positive blood microscopy	0.52	0.03–1.3
*β*_TS_	Log-odds ratio of positive blood microscopy per unit increase in natural logarithm of parasite blood concentration	1.2	0.9–1.7

Bayesian credible intervals (BCI) are defined as the 2.5th and 97.5th percentiles of the posterior sample for each parameter. All estimates of variation (including measurement error) are expressed in terms of standard deviations on the natural logarithmic scale, unless specified otherwise. For information on the assumed prior distributions and a detailed description of the statistical model, see S1 Text and Table S1 within it.

**Fig 2:**
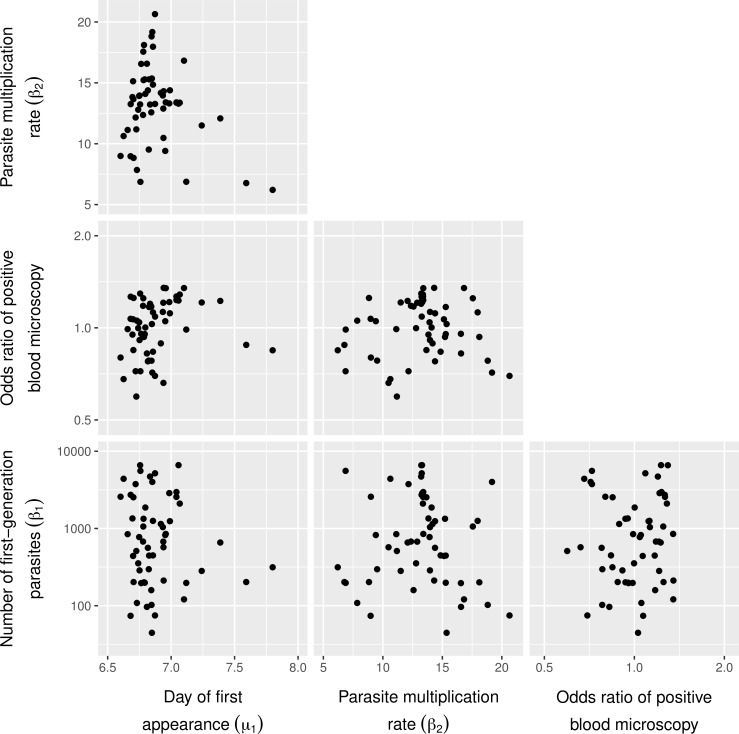
Matrix scatter plot of random effects for sources of inter-individual variation. Random effects (x-axes and y-axes) pertain to the average time of appearance of first generation blood parasites, the number of first cycle parasites, the multiplication rate of blood parasites, and the log-odds ratio of an individual having parasites detected during blood microscopy, adjusted for predicted parasite levels. Within each panel, each bullet represents the point estimate for one CHMI volunteer (*N* = 56), based on the mean of 8000 draws from the posterior.

In Fig 2, we illustrate the correlation between time of appearance of first generation blood parasites, peak concentration in blood of first generation parasites, the parasite multiplication rate, and the relative odds of an individual having positive blood microscopy. As there was no clear correlation pattern between these four individual-level parameters, we did not further explicitly model their joint distribution (i.e. we assume they are independently distributed when simulating data for power calculations).

**Fig 3:**
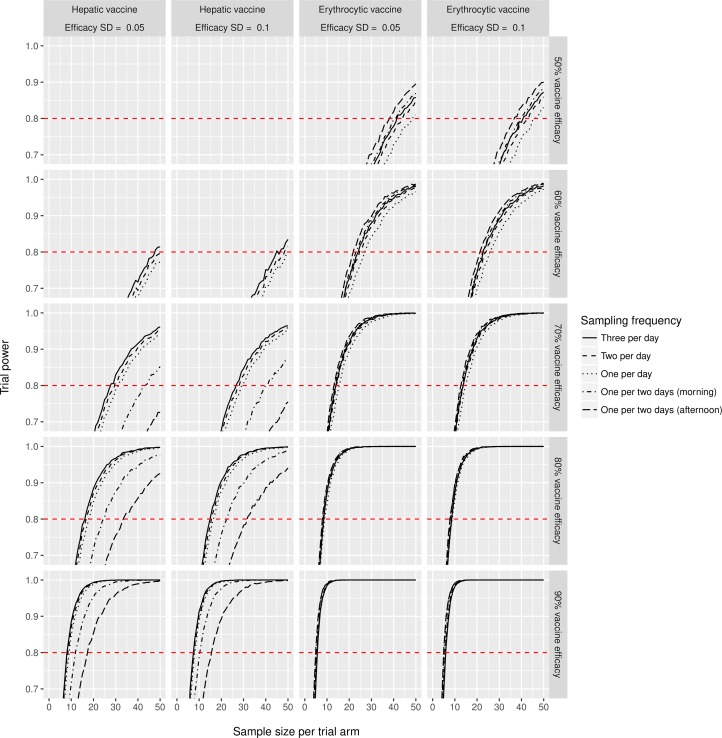
Simulated vaccine trial power to detect a statistically significant difference between an intervention and control group (T-test assuming unequal variances, setting *α* = 0.05). Simulations were performed for each combination of vaccine type (erythrocytic or hepatic), efficacy (50%, 60%, 70%, 80%, or 90% reduction in first-generation parasite loads or parasite multiplication rate), variation in efficacy between individuals (standard deviation or SD), and the frequency of blood samples taken: one per two days (8am or 4pm), or one (8am), two (8am, 4pm), or three (8am, 4pm, 10pm) per day. Power calculations for a wider range of vaccine efficacy (30%–95%) can be visualized with the graphical user interface in S2 File.

Next, we used the model to simulate ten thousand repeated data sets for each of various hypothetical vaccine trial designs. In each simulated vaccine trial, we define vaccine efficacy as the relative reduction in total number of released merozoites induced by a hepatic vaccine, or the relative reduction in parasite growth rate induced by an erythrocytic vaccine. To explicitly account for previously excluding one out of 57 original study subjects, we allowed each individual in the simulated vaccine trials to be dropped with a 1/57 probability (which turned out to be of little consequence for power estimates). Fig 3 illustrates how trial power increases with the number of individuals per trial arm, allowing one to deduce the required number of individuals for a desired power level. Study power is most dependent on the number of individuals in each treatment arm; in contrast, inter-individual variation in vaccine efficacy and the number of blood samples taken per day matter relatively little for study power. Due to high inter-individual variation in the number first-generation parasites, hepatic vaccine trials required significantly more study subjects than erythrocytic vaccine trials. For instance, over 30 individuals per trial arm are needed to achieve 80% study power when hepatic vaccine efficacy is 70% or lower. S2 File provides a graphical user interface to look up power of all simulated vaccine trial settings. The effective probability of a Type 1 error when using T-tests (assuming unequal variances and setting *α* = 0.05) was approximately 5% and even somewhat lower for small sample sizes (S3 Fig), which confirmed the validity of T-tests for identifying a difference between trial arms.

S4 Fig also depicts the association between trial power and number of individuals per treatment arm (like Fig 3), but under the assumption that inter-individual variation in peak first-generation parasite density in terms of SD is halved (e.g. by using needle-injected instead of mosquito-based CHMI). This figure illustrates how such a reduction in inter-individual variation may reduce the number of individuals required for hepatic vaccine trials. For instance, only 11 to 15 individuals per trial arm (depending on number samples per day) would be needed to achieve 80% study power when hepatic vaccine efficacy is 70%, instead of around 30 or more when using mosquito-based CHMI (Fig 3).

For comparison, we further repeated the power analysis using the log-linear sine model [22], which we also improved to explicitly capture censored observations and inter-individual variation in slope, intercept, and timing of the log-linear sine curve. Table S2 in S1 Text summarizes the assumed prior distributions and posterior parameter estimates for the sine model; S5 Fig depicts the model fit to the data. The log-linear sine model resulted in a higher estimate of the parasite growth rate than our non-linear model: 4.80 per day, or 17.9 per parasite cycle (*vs*. 11.8 per cycle), assuming a parasite cycle duration of 1.84 days (as estimated by the non-linear model). Further, the sine model resulted in a higher estimate of qPCR measurement error (1.22 *vs*. 0.98 on the natural logarithmic scale, or RSD of 155% *vs*. 129%), which is an indication of the sine model providing an inferior fit to the data compared to the non-linear model, as it attributed some of the temporal variation in the data to (random) measurement error. As a result, the sine model produced more optimistic estimates of study power than our non-linear model, especially for hepatic vaccines (S6 Fig).

## Discussion

We present robust power analyses for malaria vaccine trials based on controlled human malaria infection (CHMI) experiments, using a Bayesian non-linear model for blood parasite kinetics. Our model adequately accounts for the cyclical nature of *P*. *falciparum* concentrations in the host blood, based on robust estimates of biological parameters underlying CHMI. Our study supersedes earlier models and power calculations: we prevent overly optimistic estimates of trial power by directly modeling the biological processes behind the cyclical patterns in CHMI to best capture temporal patterns in the data, and by jointly and robustly estimating all model parameters in a Bayesian hierarchical model framework. Furthermore, we avoid excessive weighing of outliers in data simulation, appropriately model the impact of detection limits, and account for five important sources of variation between individuals: time of first parasite appearance, first cycle amplitude, parasite multiplication rates, probability of positive blood microscopy (and consequent termination of the experiment), and vaccine efficacy. This makes our model a more suitable tool for power calculations of CHMI-based vaccine trials than previous approaches to date.

Our study confirms that for vaccine trial power, inter-individual variation in vaccine efficacy and the number of blood samples taken per day matter relatively little, and that erythrocytic vaccine trials require significantly fewer study subjects than hepatic vaccine trials [23]. Importantly, our analyses suggest that loss of only a few study subjects (e.g. due to experimental failure) may lead to drastic loss of trial power, unless the trial power is already close to 100%, which is important to consider during trial design. For instance, if the anticipated efficacy of a hepatic vaccine is 80% (reduction in number of first-generation parasites), 15 people per treatment arm will provide just over 80% power to detect this level of efficacy, but drop out of even one study subject per treatment arm may diminish the trial power below 80%. Further, our results suggest that a 50% reduction in the inter-individual variation of the number of first-generation parasites (e.g. by using needle-injected instead of mosquito-based CHMI) may result in a substantial reduction in the number of individuals required per trial arm, especially so for hepatic vaccine trials (reductions up to 50%). These estimates will be further refined based on forthcoming data from needle-injected CHMI experiments.

The average multiplication rate of *P*. *falciparum* parasites estimated by our non-linear model is very similar to earlier estimates [22,24,25] or somewhat higher [21,23,26]. However, our results do not confirm an earlier report by Sheehy *et al*. of negative correlation between the parasite multiplication rate and the peak concentration of first generation parasites in mosquito-based CHMI [13], and suggest that this previous finding should be revisited. Possibly, Sheehy *et al*. overestimated the multiplication rate in study subjects with low initial parasite loads (two parasites per mL) as typically, parasite loads below the detection limit for such individuals are set to some arbitrary low value (e.g. half the value of the detection limit[26]) while ignoring measurement error (i.e. true levels may be above the detection limit). If Sheehy *et al*. had left out observations with low initial parasite loads (<10 Pf/mL) from their analysis, very little correlation would have remained.

The parameter estimates from our study are intended for power calculations for phase 1/2 hepatic and erythrocytic vaccine trials using CHMI of malaria-naive individuals with NF54 or similar strains (i.e. first-time infections only), and using qPCR to monitor parasitaemia levels. To avoid variation due to differences between labs and strains, future vaccine trials using CHMI should either be performed in a single lab using a single strain (as was the case for the data used in this study), or in case of multi-center trials, each lab should cover each trial arm to allow within-lab comparisons. Of course, the validity of power estimates based on our model relies on understanding one’s potential vaccine efficacy in terms of its impact on the number of first generation blood parasites and the parasite replication rate at the time of challenge (in contrast to impact on clinical outcomes such as time until positive blood microscopy or duration of sterile protection). The potential vaccine efficacy in a power calculation is ideally based on a target product profile (TPP) defined as part of a vaccine development process. We therefore recommend that TPPs are defined not just in terms of clinical outcomes but also in terms of impact of a vaccine on parasite growth. The link between impact on parasite growth and clinical outcomes in healthy volunteers and inhabitants of endemic areas is a topic of ongoing research. Based on our study in health volunteers, however, we can say that to achieve sterile protection in healthy volunteers, an erythrocytic vaccine would have to reduce the parasite multiplication rate by at least 92% (1 − 1/*β*_2_, where *β*_2_ is the parasite multiplication rate per cycle).

Further, our estimates for biological parameters can be used as prior information (in a Bayesian framework) in studies that aim to estimate biological and/or vaccine efficacy parameters. If there is reason to believe that the study population is somehow different from the population covered by the current study (e.g. a different parasite replication rate is expected), it is important that a sensitivity analysis be performed by incrementally diluting the relevant prior information (i.e. increasing prior variance on the population mean and variance of e.g. replication rates) so that the prior information becomes weaker and the model is relatively more informed by the data at hand.

Here, we predict that mosquito-based CHMI vaccine trials may require several tens of volunteers per trial arm, especially if the expected vaccine efficacy is under 70% (in terms of impact on patterns in parasite growth) and/or if a hepatic vaccine is being tested. In our experience and as reported in literature [27], CHMI experiments are typically executed with 5–8 volunteers at a time, or in several batches of that size. Working with larger groups is impractical and expensive because many volunteers will turn positive for malaria infection on the same day, requiring more attending physicians and lab personnel. However, future vaccine candidates should preferably have an impact on parasite growth that is much higher than 70%, which for erythrocytic vaccines can be demonstrated with 80% power using only 5 to 15 volunteers per trial arm. Achieving similar study power for hepatic vaccine trials with the same number of volunteers might be made possible by the use of needle-based CHMI.

It has been argued that the non-linear model that we build on here [21] is unnecessarily complex as simpler models may yield very similar estimates of relevant biological parameters, and requires the estimation of vaccine-irrelevant parameters and the assumption that some of those parameters are fixed [26]. We argue that log-linear models (a straight line through the log-transformed parasite concentrations [24]) and sine models (log-linear model in which the slope is multiplied with a sine function [22,28]) are too simple and require assumptions that clearly contradict the data (linearity of data; data before first parasite generation are ignored; parasite cycles follow a symmetric, sinusoid pattern), making them less fit for power calculations. In power analyses, it is imperative to consider uncertainty in all relevant parameters (like we do here) [29], including the processes leading to censoring of observations below the detection limit and termination of an experiment in case of positive blood microscopy. In this study, we demonstrate that the log-linear sine model (even after properly accounting for censoring and variation between individuals) results in higher power estimates than our non-linear model, which we attribute to the fact that the sine model does not capture temporal patterns in CHMI data as well as our non-linear model, as indicated by the sine model’s higher estimate for qPCR measurement error. The bootstrapping approach to power calculation used by Roestenberg *et al*. [23] is a major improvement over log-linear and sine models, but it is based on the assumption that the empirical distribution of data observed so far is representative of the distribution in the population, which may cause it to assign excessive weight to outliers in power calculations, especially when based on small datasets. Rather, our model, with its hierarchical design, shrinks such outliers towards the population mean when estimating population-level parameter values, while retaining the ability to simulate such outliers by chance. Because we more robustly quantify parameter uncertainty, our power estimates are more realistic and dictate higher required sample sizes compared to those by Roestenberg *et al*. [23] Further, in the current study, we relaxed the assumption of fixing parameters by jointly estimating all parameters in a Bayesian framework.

A possible limitation in the application of our model for mosquito-based CHMI data, is the assumption that first-generation malaria parasites appear in the blood in a single wave (an assumption shared with the sine model [22,28]). Obviously, this was not the case for subjects in whom no clear cyclical pattern in parasitaemia levels was visible. In this respect, CHMI data based on needle-infected PfSPZ may provide less noisy data than mosquito-based CHMI [12,13], and may even result in fewer asynchronous parasite cycles, although this remains to be evaluated. In short, like all other statistical models [22–24,28], our model relies on assumptions that are not always fulfilled; nevertheless, our model best approximates and quantifies uncertainty in the biological mechanisms behind CHMI and is therefore the most suitable for performing power calculations.

As already mentioned, only the RTS,S vaccine has shown promising results so far (30%–65% protection against clinical malaria) [2–6], with a recent large, multi-center phase three trial showing 45.7% protection against clinical malaria in infants and children aged 5–17 months over a period of 18 months after three vaccine doses [7]. Unfortunately, RTS,S vaccine trials have only used blood microscopy to evaluate infection levels in trial participants so far, which does not allow evaluation of vaccine impact in terms of the effect on parasite growth patterns (and thus a link with our power analyses). This obstacle may be readily overcome by the use of qPCR to monitor infection levels in Phase 1/2 studies, which may also help to reduce study sample sizes.

In conclusion, to maximize the probability of identifying effective candidate malaria vaccines, and to keep the risk of severe adverse events and the number of invasive procedures to a minimum, it is important to perform power analyses. With this study, we provide robust power estimates for malaria vaccine trials using mosquito-based CMHI, superseding previous models and power analyses. Given the simulation-based nature of our approach, it is straightforward to implement more complicated assumptions for future vaccine trials. Last, our model suggests that using needle-injected instead of mosquito-based CHMI may improve the power of hepatic vaccine trials.

## Materials and Methods

### Data and study population

We used data from 57 volunteers participating in 8 different sporozoite challenge trials at Radboud University Medical Center (RUMC, Nijmegen, The Netherlands) from 1999 to 2011 (Table 2) [14,21,30–35]. The data set included subjects from immunological studies (n = 20), infectivity controls from immunization trials (n = 18), and non-protected subjects from a malaria vaccine trial (n = 19). Volunteers were challenged by bites of 4–7 (n = 20) or 5 infected mosquitoes (n = 37) for 10 minutes; the number of bites was unknown as exposure to mosquito bites took place in the dark (under a cloth), and not all individuals developed a clearly visible skin reaction to every bite. Mosquitoes were laboratory-reared and infected with the NF54 strain of Pf. Presence of sporozoites in mosquitoes was confirmed by salivary gland dissection. Trial subjects were followed 2–3 times daily from day 5 after challenge until 3 days after antimalarial curative treatment. At every visit, blood samples were collected and assessed for presence of parasites by microscopy (threshold 4 parasites/μL) and quantified by qPCR (detection limit 20 or 200 parasites/mL) [14]. Ethical approval was obtained for each trial separately from the RUMC institutional review board and/or for some trials from the Dutch Central Committee on Research Involving Human Subjects (0004–00900, 0011–0262, 2001/203, 2002/170, NCT00442377, NCT00757887, NCT00509158, NCT01002833, NCT01236612).

**Table 2:** Study characteristics.

Study	Data marker	Type*	Number of human volunteers	Number of mosquitoes per volunteer	Parasite strain	qPCR Detection limit	Citation
0004–00900	EHMI-2	CHMI	5	4–7	NF54	20 Pf/ml	[14]
0011–0262	EHMI-3	CHMI	5	4–7	NF54	20 Pf/ml	[21]
2001/203	EHMI-4	CHMI	5	4–7	NF54	20 Pf/ml	[21]
2002/170	EHMI-5	CHMI	5	4–7	NF54	20 Pf/ml	[30]
NCT00442377	EHMI-8A	CIS	5	5	NF54	20 Pf/ml	[31]
NCT00757887	EHMI-8B	CIS	5	5	NF54	20 Pf/ml	[32]
NCT00509158	LSA3	CIS	18	5	NF54	200 Pf/ml	Unpublished
NCT01002833	TIP-1	CHMI	4	5	NF54	20 Pf/ml	[33]
NCT01236612	ZonMw	CIS	5	5	NF54	20 Pf/ml	[34,35]

*CHMI: Controlled Human Malaria Infection; CIS: Controls of an Immunization Study

### Statistical model

We modeled parasite kinetics in a novel Bayesian non-linear statistical model, which is based on a previous, simpler model for cyclical patterns in CHMI [21]. The model predicts parasite concentration in the blood as measured by qPCR as a function of days since infection, mimicking successive cycles of appearance and disappearance (sequestration) of blood parasite generations. The model parameters capture the following biological processes: the total number of first generation blood parasites per mL blood (*β*_1_); the blood parasite multiplication rate, i.e. the number of next generation parasites per current generation parasite (*β*_2_); the average time from inoculation to appearance of first generation blood parasites (*μ*_1_); the average duration of the blood parasite stage (*μ*_2_); the average time from parasite sequestration (i.e. when a parasite cannot be detected in the blood) to appearance of next generation parasites (*μ*_3_); and the standard deviation of time of appearance and disappearance of individual blood parasites from a given generation, which represents the rate at which parasite concentrations change (*σ*_1_, lower values mean higher rate). We assumed that qPCR measurement error follows a lognormal distribution.

We extended the original model [21] with regard to the following points. We allowed parasite kinetics to vary between individuals by including random effects for model parameters *β*_1_, *β*_2_, and *μ*_1_. Given that the nine CHMI experiments (Table 2) were performed at the same lab, using the same strain, and were performed by the same person (CCH) on the same PCR machine, we assumed that inter-study variation is negligible relative to inter-individual variation (i.e. no random intercept for study). Observations below the detection limit of the qPCR (i.e. censored observations) were explicitly modeled, rather than assuming a value equal to half the detection limit [21,26] (which would introduce artificial information) or leaving out such observations altogether [28] (which would ignore information in the data). We further jointly modeled the probability of detecting parasites through blood microscopy as a function of the predicted parasite load (i.e. before measurement error or censoring), using a standard hierarchical logistic regression model (random intercept for individuals).

Model parameters were jointly estimated in a Bayesian framework, giving several advantages over previously applied classic (frequentist) approaches [21,25,26]. The Bayesian approach allowed simultaneous estimation of all model parameters and the associated uncertainty (including the model parameters for positive blood microscopy), without the need to fix a subset of parameters. Furthermore, the Bayesian framework allowed for exact rather than approximate inferences based on normality assumptions. See S1 Text for a detailed description of the statistical model and the parameter estimation procedure. Model parameter estimates are summarized in terms of posterior mean and a 95%-Bayesian credible interval (95%-BCI), which we defined as the 2.5^th^ and 97.5^th^ percentiles of the posterior samples for each parameter.

### Power analysis

We performed power calculations for erythrocytic and hepatic vaccine trials, using combinations of presumed mean vaccine efficacy (30%, 40%, 50%, 60%, 70%, 80%, 90%, or 95% reduction in number of first-generation parasites or parasite multiplication rate), inter-individual variation in vaccine effect (beta distribution with standard deviation 0.05 or 0.10), number of study participants in each group, and the blood sampling frequency: one (8am), two (8am, 4pm), or three (8am, 4pm, 10pm) per day, or once every two days (even days), either in the morning (8am) or afternoon (4pm). For each combination of assumptions, we simulated ten thousand repeated vaccine trials, using one posterior draw of model parameters to generate one set of trial data. We explicitly simulated the probability of blood microscopy turning positive (and consequent termination of the experiment) as a function of predicted blood parasite concentrations to arrive at the most realistic individual time series possible. For each repeated vaccine trial, all individual-level random effects were drawn independently from each other (i.e. we did not re-use the random effects estimated from the data). The qPCR detection limit was set to 20 parasites per mL blood (i.e. the current practice at Radboud University Medical Center, Nijmegen, The Netherlands).

Because the Bayesian non-linear model used in this study requires a substantial amount of data (i.e. at least two, preferably three samples per day), each simulated vaccine trial was analyzed using simple frequentist statistical tests as previously described [23]. First, censored observations were set to half the value of the detection limit. Next, we categorized data by parasite cycle (days 6.5–8.5, 8.5–10.5, 10.5–12.5) and calculated the mean log-transformed blood parasite concentration per parasite cycle and individual. For hepatic vaccine trials, we compared first-cycle log-blood concentrations between vaccine and control arms with two-sided t-tests (assuming unequal variances due to potential censoring). For asexual erythrocytic vaccine trials, we calculated the differences in average log-blood concentrations between consecutive cycles for each individual, and then averaged these differences over cycles within individuals. The average differences were compared between groups, again with two-sided T-tests (assuming unequal variances due to potential censoring). The predicted power of a vaccine trial was expressed as the proportion of repeatedly simulated trials that resulted in a *p*-value equal to or lower than 0.05. The validity of using the T-test (i.e. the effective probability of a Type 1 error) was checked in a similar fashion, but by simulating vaccine trials with zero effect in both treatment arms.

To explore the potential impact of using needle-injected CHMI on vaccine trial power, we repeated the power calculations assuming that the inter-individual variation in the number of first-generation parasites is half that of mosquito-based CHMI (i.e. $\sigma_{\beta_{1},\text{needle}}=\sigma_{\beta_{1}}/2$, based on data digitally extracted from Fig 4A in Sheehy *et al*. 2013 [13]).

We further repeated the power calculation based on an analysis of the data using the simpler log-linear sine model [22], assuming that the parasite generation time is 1.84 days (as estimated by the main model). For the sake of comparison, we improved the log-linear sine model by adding random effects for starting parasitaemia levels, parasite growth rate, and timing of the parasite cycle in each individual, and explicitly modeled observations under the qPCR detection limit (see S1 Text for model details). For the purpose of the power analysis we assumed that two full parasite cycles would be observed in each individual (the log-linear sine model does not provide a prediction for termination of an experiment). Other than that, this power analysis was executed in exactly the same fashion as that based on the main analysis.

### Role of funding source

None of the funders were involved in the writing of the manuscript or the decision to submit it for publication. The authors have not been paid to write this article by a pharmaceutical company or other agency. The corresponding authors had full access to all the data in the study and had final responsibility for the decision to submit for publication.

## Supporting Information

S1 Text Detailed description of the statistical model and parameter estimation procedure. (DOCX)

S1 Fig CHMI data for all 57 individuals and parasite blood dynamics as predicted by the non-linear model. Solid lines represent the posterior mean; shaded bands represent the 2.5^th^ and 97.5^th^ percentiles of the predicted parasite concentrations, based on 8000 draws from the posterior distribution. Panel headers refer to unique identifiers for CHMI volunteers, which can also be found in the data (S1 File).
(PDF)

S2 Fig Association between parasite concentration in the blood and blood smear positivity. Vertical bars represent observed data on blood smear positivity (0/1) and parasite concentration. Note that the predicted probability of blood smear positivity is based on model-predicted parasite concentration; sigmoid lines represent predicted probabilities for different individuals and are each based on the mean of 8000 posterior draws.
(PDF)

S3 Fig Effective probability of Type 1 error when using T-tests to analyse CHMI data, assuming unequal variances and setting *α* = 0.05 (two figures over two pages). (PDF)

S4 Fig Vaccine trial power calculated in the same fashion as for Fig 3, but assuming that inter-individual variation in the number of first-generation parasites is half that of mosquito-based CHMI. (PDF)

S5 Fig CHMI data for all 57 individuals and parasite blood dynamics as predicted by the sine model. Solid lines represent the posterior mean; shaded bands represent the 2.5^th^ and 97.5^th^ percentiles of the predicted parasite concentrations, based on 8000 draws from the posterior distribution. Panel headers refer to unique identifiers for CHMI volunteers, which can also be found in the data (S1 File).
(PDF)

S6 Fig Vaccine trial power calculated in the same fashion as for Fig 3, but using the log-linear sine model, assuming that two full parasite cycles are observed in all individuals. (PDF)

S1 File CHMI data used in the analyses (comma-separated file). (CSV)

S2 File Graphical user interface to visualize power for different vaccine trial designs and to determine the minimal required sample size for user-defined minimum power (excel spreadsheet). (XLSX)
