# Supplementary material for: The Power of Malaria Vaccine Trials Using Controlled Human Malaria Infection
Source: PLoS Comput Biol. 2017 Jan 12;13(1):e1005255. doi: 10.1371/journal.pcbi.1005255 (PMC5230743; doi:10.1371/journal.pcbi.1005255)
Supplement: S1 Text — (DOCX) [file pcbi.1005255.s001.docx]

Supplemental Text S1: Detailed description of the statistical model and parameter estimation procedure

Luc E. Coffeng,^1*^ Cornelus C. Hermsen,^2^ Robert W. Sauerwein,^2^ Sake J. de Vlas^1^

^1^ Department of Public Health, Erasmus MC, University Medical Center Rotterdam, PO Box 2040, 3000 CA Rotterdam, The Netherlands

^2^ Department of Medical Microbiology, Radboud University Medical Center, PO Box 9101, 6500 HB, Nijmegen, The Netherlands

^*^ Corresponding author: [l.coffeng@erasmusmc.nl](mailto:l.coffeng@erasmusmc.nl)

# Statistical model

## Non-linear model for parasite kinetics

The non-linear model used in our main analysis predicts parasite concentration ($y_{\text{Pf}})$ in the blood as a function of days ($t$) since infection, mimicking $J$ successive cycles of appearance and disappearance (sequestration) of blood parasite generations:

| $y_{\text{Pf}}\left( t \right)=\beta_{1}\sum_{j=1}^{J} \beta_{2}^{j-1}\left[ \phi\left( t,M_{j1},\Sigma_{j1} \right)-\phi\left( t,M_{j2},\Sigma_{j2} \right) \right]$ | (1) |
| --- | --- |

Here, $\beta_{1}$ represents the peak number of first generation blood parasites per mL blood; $\beta_{2}$ is the blood parasite multiplication rate (number of next generation parasites per current generation parasite); and $\phi$ is the cumulative normal distribution function. $M_{j1}$ and $M_{j2}$ are the average time of appearance and disappearance of blood parasites of the $j$-th generation, respectively; and $\Sigma_{j1}$ and $\Sigma_{j2}$ are the standard deviations in appearance and disappearance of blood parasites of the $j$-th generation, respectively. For computational speed, we approximate $\phi\left( t,M,\Sigma\right)$ with the function $\phi'\left( x \right)=\text{logit}^{-1}(0.07056x^{3}+1.5976x)$, where $x=\left( t-M \right)/\Sigma$. The maximum absolute error of this approximation is 0.00014. $M_{j1}$, $M_{j2}$, $\Sigma_{j1}$ and $\Sigma_{j2}$ are defined as:

| $M_{j1}=\mu_{1}+\left( j-1 \right)\mu_{2}+\left( j-1 \right)\mu_{3}$ | (2) |
| --- | --- |
| $M_{j2}=\mu_{1}+j\mu_{2}+\left( j-1 \right)\mu_{3}$ | (3) |
| $\Sigma_{j1}=\sqrt{\sigma_{1}^{2}+\left( j-1 \right)\sigma_{2}^{2}+\left( j-1 \right)\sigma_{3}^{2}}$ | (4) |
| $\Sigma_{j2}=\sqrt{\sigma_{1}^{2}+j\sigma_{2}^{2}+\left( j-1 \right)\sigma_{3}^{2}}$ | (5) |

Here, $\mu_{1}$ is the average time from inoculation to appearance of first generation blood parasites, $\mu_{2}$ is the average duration of the blood parasite stage, and $\mu_{3}$ is average time from parasite sequestration (i.e. when a parasite cannot be detected in the blood) to appearance of next generation parasites. The standard deviations of each of these three durations (within a single generation of parasites) are represented by $\sigma_{1}$, $\sigma_{2}$, and$\sigma_{3}$, respectively.

To account for correlation of repeated measurements within individuals over time, we included random effects for model parameters $\mu_{1}$, $\beta_{1}$, and $\beta_{2}$. In other words, we assumed that the average time of appearance ($\mu_{1})$ and size ($\beta_{1}$) of the first generation of blood parasites varied between individuals, as well as the parasite multiplication rate ($\beta_{2}$). The random effects for each of these parameters were assumed to follow independent lognormal distributions with unknown population mean and standard deviation. Note that for convenience, the individual subscript *i* was dropped for these random effects in equations 1-5.

Measurement error of the qPCR was assumed to follow a lognormal distribution. Observations below the detection limit of the qPCR were explicitly modeled, rather than assuming a value equal to half the detection limit [1,2] (which would introduce artificial information into the data) or leaving out such observations altogether [3] (which would ignore information in the data). For instance, if the model predicted a parasite concentration of 18 parasites per mL blood for an observation that was below the detection limit of 20 parasites per mL blood, the likelihood was calculated as the cumulative probability of observing a value under 20, given a log-normal distribution with geometric mean 18 and some variance $\sigma_{y}^{2}$ representing the qPCR measurement error (which was estimated from the rest of the data above the detection limit).

We further predicted the probability $P(\text{TS}_{i,t}^{+})$ of detecting parasites through blood microscopy in individual *i* at time *t* as a function of the log-transformed parasite load ${y_{\text{Pf}}\left( t \right)}_{i}$ (i.e. as predicted by the model described above, without measurement error or censoring), using a hierarchical logistic regression model:

| $P(\text{TS}_{i,t}^{+})=1/\left( 1+exp(-\left( \alpha_{\text{TS}}+\beta_{\text{TS}}\cdot\ln({y_{\text{Pf}}\left( t \right)}_{i})+\epsilon_{i} \right) \right)$ | (6) |
| --- | --- |
| $\epsilon_{i}\sim\text{Normal}\left( 0, \sigma_{\text{TS}} \right)$ | (7) |

The error term $\epsilon_{i}$ represents the variation between individuals in log-odds of detecting parasites through blood microscopy, adjusted for predicted log-parasite loads.

## Log-linear sine model for parasite kinetics

The log-linear sine model [4] describes expected parasite concentration ($y_{\text{Pf}})$ in the blood as a function of days ($t$) since infection as follows:

| $y_{\text{Pf}}\left( t \right)=\beta_{1}\cdot\beta_{2}^{t}\cdot e^{\left( \alpha\cdot\sin\left( \frac{2\pi t}{\mu_{23}}-\mu_{1} \right) \right)}$ | (8) |
| --- | --- |

which is equivalent to:

| $\log\left( y_{\text{Pf}}\left( t \right) \right)=\log\left( \beta_{1} \right)+t\cdot\log\left( \beta_{2} \right)+\alpha\cdot\sin\left( \frac{2\pi t}{\mu_{23}}-\mu_{1} \right)$ | (9) |
| --- | --- |

Here, $\beta_{1}$ represents the hypothetical number of blood parasites per mL blood at time zero; $\beta_{2}$ is the blood parasite multiplication rate per day (N.B. not per cycle); $\mu_{1}$ is an offset for the timing of the sine pattern relative to time zero, expressed in radians and bounded by an arbitrary interval of length $2\pi$; last, $\mu_{23}$ is the period of the sine pattern, and represents the parasite generation time.

To be able to compare power calculations based on the sine model with those based on the non-linear model, we also extended the sine model with random intercepts for $\beta_{1}$, $\beta_{2}$, and $\mu_{1}$ in a similar fashion as the non-linear model. Because parameter $\mu_{1}$ is naturally bounded by an arbitrary interval of length $2\pi$, the prior distribution should be circular such that $\text{P}\left( \mu_{1} \right)=\text{P}\left( \text{mod}\left( \mu_{1},2\pi\right) \right)$, like the Von Mises distribution, which is a close approximation of the circular normal distribution. Further, because the random effects for $\beta_{1}$ and $\beta_{2}$ were correlated (unlike in the non-linear model, which we attribute to the sine model’s inferior fit to the data), we included correlation parameter $\rho_{\beta_{12}}$ so we could reproduce this correlation in the power analyses. Measurement error and censoring of observations under the detection limit were modeled in exactly the same fashion as in the non-linear model.

# Parameter estimation

Model parameters were jointly estimated in a Bayesian framework, giving several advantages over previously applied classic (frequentist) approaches [1,2,5]. The Bayesian approach allowed simultaneous estimation of all model parameters and the associated uncertainty (including the model parameters for positive blood microscopy), without the need to fix a subset of parameters. Furthermore, the Bayesian framework allowed for exact rather than approximate inferences based on normality assumptions.

For efficient parameter estimation, we defined mildly informative proper priors for all unknown parameters (Table S1 at the end of this document). To anchor the cyclical pattern, we assumed that for each individual all observations up to the first above the qPCR detection limit consisted of first generation parasites only. Because parameters $\mu_{2}$ and $\mu_{3}$ were highly correlated, we re-parameterised them as parameters $\mu_{23}$ (total duration of a parasite generation, equivalent to $\mu_{2}+\mu_{3}$) and $\alpha_{23}$ (range 0–1), such that $\mu_{2}=\mu_{23}\alpha_{23}$ and $\mu_{3}=\mu_{23}\left( {1-\alpha}_{23} \right)$. Furthermore, we set parameters $\sigma_{2}$ and $\sigma_{3}$ to zero; these parameters represent desynchronisation of parasite cycles over time, and are only important for observation after many cycles when the parasite generations progressively overlap. Because the values of these parameters are probably about 10-fold smaller than that of $\sigma_{1}$ [1], and most of the data only covered one to two parasite cycles, we decided to set these parameters at zero in the current analysis.

By means of Hamiltonian Monte Carlo techniques [6], we sampled from the joint posterior distribution of parameter values, using a No-U-Turn Sampler (NUTS) [7], as implemented in the C++ library Stan (version 2.7.0) [8]. Stan was called from R (version 3.2.0) [9], using the package *rstan* (version 2.7.0) [8]. The posterior was sampled in eight parallel Markov chains. Each chain consisted of 2000 samples, of which the first 1000 were used for adaptation of the NUTS algorithm and discarded. To validate the model fitting algorithm, we checked and confirmed that it could recover simulated data generated by the model itself.

Table S1 summarises prior and posterior parameter estimates. For all parameters but the fraction of generation time spent as blood stage ${(\alpha}_{23}$), the data provided ample information in addition to the prior, as can be seen from the large differences between the prior and posterior 95%-BCIs. For parameter $\alpha_{23}$, which equates to $\mu_{2}/\left( \mu_{2}+\mu_{3} \right)$, the posterior was almost identical to the prior, indicating that the data provided little to no information, which was to be expected given the blood sampling frequency (two to three times per day). In a sensitivity analysis, we doubled the amount of information in the prior for $\alpha_{23}$ by setting it to $\text{Beta}\left( 130, 70 \right)$ and found that all posterior estimates for other parameters remained identical. Reducing the amount of information in the prior for $\alpha_{23}$ led to the model being poorly identifiable (multimodality of the posterior). Therefore, we used the $\text{Beta}\left( 65, 35 \right)$ as a minimally informative prior in our final analysis.

# References

1. Hermsen CC, de Vlas SJ, van Gemert GJA, Telgt DSC, Verhage DF, et al. (2004) Testing vaccines in human experimental malaria: statistical analysis of parasitemia measured by a quantitative real-time polymerase chain reaction. *Am J Trop Med Hyg* **71**: 196–201.

2. Douglas AD, Edwards NJ, Duncan CJA, Thompson FM, Sheehy SH, et al. (2013) Comparison of modeling methods to determine liver-to-blood inocula and parasite multiplication rates during controlled human malaria infection. *J Infect Dis* **208**: 340–345.

3. Simpson JA, Aarons L, Collins WE, Jeffery GM, White NJ (2002) Population dynamics of untreated *Plasmodium falciparum* malaria within the adult human host during the expansion phase of the infection. *Parasitology* **124**: 247–263.

4. Bejon P, Andrews L, Andersen RF, Dunachie S, Webster D, et al. (2005) Calculation of liver-to-blood inocula, parasite growth rates, and preerythrocytic vaccine efficacy, from serial quantitative polymerase chain reaction studies of volunteers challenged with malaria sporozoites. *J Infect Dis* **191**: 619–626.

5. Spring MD, Cummings JF, Ockenhouse CF, Dutta S, Reidler R, et al. (2009) Phase 1/2a study of the malaria vaccine candidate apical membrane antigen-1 (AMA-1) administered in adjuvant system AS01B or AS02A. *PLoS One* **4**: e5254.

6. Neal RM (2011) MCMC Using Hamiltonian Dynamics. In: Brooks S, Gelman A, Jones GL, Meng X-L. *Handbook of Markov Chain Monte Carlo*. Chapman and Hall/CRC. pp. 113–162.

7. Hoffman M, Gelman A (2014) The No-U-Turn Sampler: Adaptively Setting Path Lengths in Hamiltonian Monte Carlo. *J Mach Learn Res* **15**: 30.

8. Stan Development Team (2015) Stan: A C++ Library for Probability and Sampling, Version 2.7.0.

9. R Development Core Team (2013) R: A Language and Environment for Statistical Computing.

Table S1. Prior distributions and posterior parameter estimates for the non-linear model. All scale-parameters ($\boldsymbol{\sigma}$) of prior distributions are defined in terms of standard deviations. Half-normal priors for strictly positive parameters are indicated with a ‘+’ superscript.

|  |  |  |  | Posterior estimates | |
| --- | --- | --- | --- | --- | --- |
| Parameter | Interpretation | Prior distribution | Prior 95%-BCI | Mean | 95%-BCI |
| $\beta_{1}$ | Geometric mean number of first generation parasites per mL blood | $\text{Log-normal}\left( \ln\left( 850 \right),1 \right)$ | 120 – 6043 | 635 | 406 – 945 |
| $\sigma_{\beta_{1}}$ | Inter-individual variation in natural logarithm of number of first generation parasites per mL blood | $\text{Normal}^{+}\left( 0, 2 \right)$ | 0.6 – 4.9 | 1.40 | 1.12 – 1.74 |
| $\beta_{2}$ | Geometric mean multiplication rate of parasites per cycle | $\text{Log-normal}\left( \ln\left( 7.5 \right),1 \right)$ | 1.1 – 53.2 | 11.8 | 9.0 – 15.3 |
| $\sigma_{\beta_{2}}$ | Inter-individual variation in natural logarithm of multiplication rate of parasites | $\text{Normal}^{+}\left( 0, 0.4 \right)$ | 0.01 – 0.90 | 0.47 | 0.27 – 0.70 |
| $\mu_{1}$ | Geometric mean time of appearance of first generation parasites (days) | $\text{Log-normal}\left( \ln\left( 7 \right),0.05 \right)$^a^ | 6.4 – 7.7 ^a^ | 6.87 | 6.76 – 6.99 |
| $\sigma_{\mu_{1}}$ | Inter-individual variation in natural logarithm of time of appearance of first generation parasites | $\text{Normal}^{+}\left( 0, 0.3 \right)$ | 0.01 – 0.67 | 0.036 | 0.025 – 0.046 |
| $\sigma_{1}$ | Within-individual variation in time of appearance of first generation parasites (days) | $\text{Normal}^{+}\left( 0, 0.2 \right)$ | 0.01 – 0.45 | 0.24 | 0.20 – 0.28 |
| $\mu_{23}$ | Parasite generation time (days) | $\text{Log-normal}\left( \ln(2), 1 \right)$ | 0.3 – 14.2 | 1.84 | 1.79 – 1.91 |
| $\alpha_{23}$ | Fraction of generation time spent as blood stage parasite | $\text{Beta}\left( 65, 35 \right)$ | 0.55 – 0.74 | 0.64 | 0.57 – 0.72 |
| $\mu_{2}$ | Parasite blood stage duration ($\mu_{2}=\mu_{23}\cdot\alpha_{23}$) | Derived from $\mu_{23}$ and $\alpha_{23}$ | 0.2 – 9.4 | 1.19 | 1.04 – 1.34 |
| $\mu_{3}$ | Parasite sequestration duration ($\mu_{3}=\mu_{23}\cdot\left( 1-\alpha_{23} \right)$) | Derived from $\mu_{23}$ and $\alpha_{23}$ | 0.1 – 5.1 | 0.66 | 0.53 – 0.79 |
| $\sigma_{y}$ | Measurement error in natural logarithm of parasite blood concentration. | $\text{Normal}^{+}\left( 0, 0.5 \right)$ | 0.02 – 1.12 | 0.98 | 0.90 – 1.07 |
| $\alpha_{\text{TS}}$ | Average log-odds of positive blood microscopy in case of 1 parasite per mL blood | $\text{Normal}\left( -5, 5 \right)$ | -14.8 – 4.8 | -11.3 | -15.1 – -8.4 |
| $\sigma_{\text{TS}}$ | Inter-individual variation in log-odds of positive blood microscopy | $\text{Normal}^{+}\left( 0, 2 \right)$ | 0.6 – 4.9 | 0.52 | 0.03 – 1.3 |
| $\beta_{\text{TS}}$ | Increase in log-odds of positive blood microscopy per unit increase in natural logarithm of parasite blood concentration | $\text{Normal}\left( 0, 5 \right)$ | -9.8 – 9.8 | 1.2 | 0.9 – 1.7 |
| ^a^ To prevent chains from diverging to suboptimal posterior modes, the prior for $\mu_{1}$was truncated at the values 6.0 and 8.0. Without truncation, the prior 95%-BCI for $\mu_{1}$ would have been 6.3 – 7.7, indicating that truncation mattered little for posterior inference, apart from identifying the model. | | | | | |

Table S2. Prior distributions and posterior parameter estimates for the log-linear sine model. All scale-parameters ($\boldsymbol{\sigma}$) of prior distributions are defined in terms of standard deviations. Half-normal priors for strictly positive parameters are indicated with a ‘+’ superscript.

|  |  |  |  | Posterior estimates | |
| --- | --- | --- | --- | --- | --- |
| Parameter | Interpretation | Prior distribution | Prior 95%-BCI | Mean | 95%-BCI |
| $\beta_{1}$ | Geometric mean hypothetical number of parasites per mL blood at time = 0 | $\text{Log-normal}\left( -2,5 \right)$ | 7.51e-6 – 2400 | 2.25e-3 | 5.29e-4 – 6.2e-3 |
| $\sigma_{\beta_{1}}$ | Inter-individual variation in natural logarithm of hypothetical number of parasites per mL blood at time = 0 | $\mathrm{Normal}^{+}\left( 0, 5 \right)$ | 0.2 – 11.2 | 3.22 | 2.06 – 4.52 |
| $\beta_{2}$ | Geometric mean multiplication rate of parasites per day | $\text{Log-normal}\left( \ln\left( 7.5 \right)/2,2 \right)$ | 0.054 – 138 | 4.80 | 4.12 – 5.63 |
| $\sigma_{\beta_{2}}$ | Inter-individual variation in natural logarithm of multiplication rate of parasites | $\mathrm{Normal}^{+}\left( 0, 5 \right)$ | 0.2 – 11.2 | 0.40 | 0.27 – 0.56 |
| $\rho_{\beta_{12}}$ | Correlation between $\beta_{1}$ and $\beta_{2}$ on the natural logarithmic scale | $\mathrm{Uniform}\left( -1,1 \right)$ | -0.95 – 0.95 | -0.92 | -0.97 – -0.82 |
| $\mu_{1}$ | Offset for timing of sine cycle, relative to time = 0 (radians)^a^ | $\mathrm{Uniform}\left( -2\pi,0 \right)$ | -6.13 – -0.16 | -2.44 | -2.64 – -2.24 |
| $\kappa_{\mu_{1}}$ | Inter-individual variation in timing of sine cycle (higher value means lower variation)^a^ | $\mathrm{Normal}^{+}\left( 0, 10 \right)$ | 0.4 – 22.4 | 15.5 | 6.6 – 28.6 |
| $\alpha$ | Amplitude of sine cycle | $\mathrm{Uniform}\left( 0,\infty\right)$ | 0 – $\infty$ | 0.94 | 0.79 – 1.09 |
| $\mu_{23}$ | Parasite generation time (days) | Constant: 1.84 | - | - | - |
| $\sigma_{y}$ | Measurement error in natural logarithm of parasite blood concentration. | $\mathrm{Normal}^{+}\left( 0, 5 \right)$ | 0.2 – 11.2 | 1.22 | 1.13 – 1.31 |
| ^a^ Individual timings of the sine cycle (in radians) were assumed to follow a Von Mises distribution on the interval $\left[ -2\pi,0 \right]$ with mean $\mu_{1}$ and shape $\kappa_{\mu_{1}}$. The Von Mises distribution is a circular continuous distribution on any interval of length $2\pi$ and is therefore an appropriate prior for translating a sine curve. | | | | | |
|  |  |  |  |  |  |

# Stan code for non-linear model

functions {

real log_Pf_ml_one_cycle(real time, real T1, real t2, real sigma_t1,

real M1_log) {

real pf_ml_relative;

pf_ml_relative <- (Phi_approx((time - T1) / sigma_t1) -

Phi_approx((time - T1 - t2) / sigma_t1));

return log(pf_ml_relative) + M1_log;

}

real log_Pf_ml(real time, real T1, real t2, real t3, real sigma_t1,

real M1_log, real R, int Ncyc) {

real pf_ml_relative;

pf_ml_relative <- (Phi_approx((time - T1) / sigma_t1) -

Phi_approx((time - T1 - t2) / sigma_t1));

for(i in 2:Ncyc)

pf_ml_relative <- pf_ml_relative + R^(i-1) *

(Phi_approx((time - T1 - (i-1)*(t2 + t3)) / sigma_t1) -

Phi_approx((time - T1 - i*t2 - (i-1)*t3) / sigma_t1));

return log(pf_ml_relative) + M1_log;

}

}

data {

int<lower=0> Nobs; // Number of observations

int<lower=0> Nind; // Number of individuals

int<lower=0> Ncyc; // Number of cycles to model

real<lower=0> dlimit20; // Low detection limit

real<lower=0> dlimit200; // High detection limit

int<lower=0> N_cens1_d20; // Number of censored observations before

// first positive observation (dlimit = 20)

int<lower=0> id_cens1_d20[N_cens1_d20]; // Individual to which censored observation

// pertains

vector<lower=0>[N_cens1_d20] time_cens1_d20; // Time of censored observation

vector<lower=0>[N_cens1_d20] dlimit_cens1_d20; // Lower detection limit of blood parasite

// concentrations

int<lower=0,upper=1> TS_cens1_d20[N_cens1_d20]; // Bloodsmear positivity (0/1)

int<lower=0> N_cens1_d200; // Number of censored observations before

// first positive observation (dlimit = 200)

int<lower=0> id_cens1_d200[N_cens1_d200]; // Individual to which censored observation

// pertains

vector<lower=0>[N_cens1_d200] time_cens1_d200; // Time of censored observation

vector<lower=0>[N_cens1_d200] dlimit_cens1_d200; // Lower detection limit of blood parasite

// concentrations

int<lower=0,upper=1> TS_cens1_d200[N_cens1_d200]; // Bloodsmear positivity (0/1)

int<lower=0> N_cens2_d20; // Number of censored observations after

// first positive observation (dlimit = 20)

int<lower=0> id_cens2_d20[N_cens2_d20]; // Individual to which censored observation

// pertains

vector<lower=0>[N_cens2_d20] time_cens2_d20; // Time of censored observation

vector<lower=0>[N_cens2_d20] dlimit_cens2_d20; // Lower detection limit of blood parasite

// concentrations

int<lower=0,upper=1> TS_cens2_d20[N_cens2_d20]; // Bloodsmear positivity (0/1)

int<lower=0> N_cens2_d200; // Number of censored observations after

// first positive observation (dlimit = 200)

int<lower=0> id_cens2_d200[N_cens2_d200]; // Individual to which censored observation

// pertains

vector<lower=0>[N_cens2_d200] time_cens2_d200; // Time of censored observation

vector<lower=0>[N_cens2_d200] dlimit_cens2_d200; // Lower detection limit of blood parasite

// concentrations

int<lower=0,upper=1> TS_cens2_d200[N_cens2_d200]; // Bloodsmear positivity (0/1)

int<lower=0> N_firstpos; // Number of first positive observations

int<lower=0> id_firstpos[N_firstpos]; // Individual to which first positive

// observation pertains

vector<lower=0>[N_firstpos] time_firstpos; // Time of first positive observation

vector<lower=0>[N_firstpos] pf_ml_firstpos; // Value of first positive observation

int<lower=0,upper=1> TS_firstpos[N_firstpos]; // Bloodsmear positivity (0/1)

int<lower=0> N_cyc1plus; // Number of non-censored observations after

// first positive observation + known

// bloodsmear results

int<lower=0> id_cyc1plus[N_cyc1plus]; // Individual to which non-censored

// observation after first positive

// observation pertains

vector<lower=0>[N_cyc1plus] time_cyc1plus; // Time of non-censored observation after

// first positive observation

vector<lower=0>[N_cyc1plus] pf_ml_cyc1plus; // Value of non-censored observation after

// first positive observation

int<lower=0,upper=1> TS_cyc1plus[N_cyc1plus]; // Bloodsmear positivity (0/1)

int<lower=0> N_cyc1plus_TS_NA; // Number of non-censored observations

// after first positive observation +

// unknown bloodsmear results

int<lower=0> id_cyc1plus_TS_NA[N_cyc1plus_TS_NA]; // Individual to which non-censored

// observation after first positive

// observation pertains

vector<lower=0>[N_cyc1plus_TS_NA] time_cyc1plus_TS_NA; // Time of non-censored observation

// after first positive observation

vector<lower=0>[N_cyc1plus_TS_NA] pf_ml_cyc1plus_TS_NA; // Value of non-censored observation

// after first positive observation

}

transformed data{

real dlimit20_log;

real dlimit200_log;

dlimit20_log <- log(dlimit20);

dlimit200_log <- log(dlimit200);

}

parameters {

// Time of appearance of first generation parasites

real<lower=log(6),upper=log(8)> T1_log_mu; // Average among individuals in population

real<lower=0> T1_sd; // Standard deviation among individuals in

// population

real<lower=6,upper=8> t1[Nind]; // Individual-level

real<lower=0> sigma_t1; // Standard deviation between parasites (log-

// scale)

// Parasite generation duration

real<lower=0> t23; // Total parasite generation time

real<lower=0,upper=1> t2of3; // Fraction of parasite generation time spent in ring-form

// Number of parasites in first generation

real M1_log_mu; // Average among individuals in population

real<lower=0> M1_sd; // Standard deviation among individuals in population

real m1_raw[Nind]; // Individual-level (normalized)

// Multiplication rate of parasites (per generation)

real<lower=0> R_log_mu; // Average among individuals in population

real<lower=0> R_sd; // Standard deviation among individuals in population

real<lower=0> r[Nind]; // Individual-level

// Measurement error (log-scale)

real<lower=0> sigma;

// Values of censored observations

real<upper=dlimit20_log> log_pf_ml_cens1_d20[N_cens1_d20]; // Obs before first positive

real<upper=dlimit200_log> log_pf_ml_cens1_d200[N_cens1_d200]; // Obs before first positive

real<upper=dlimit20_log> log_pf_ml_cens2_d20[N_cens2_d20]; // Obs after first positive

real<upper=dlimit200_log> log_pf_ml_cens2_d200[N_cens2_d200]; // Obs after first positive

// Logistic regression coefficients for bloodsmear positivity

real a; // intercept

real b; // slope

vector[Nind] a_raw; // random effect (normalized)

real<lower=0> a_sd; // scale of random effect

}

transformed parameters {

real<lower=0> t2; // Duration of blood stage

real<lower=0> t3; // Duration of sequestration

real m1_log[Nind]; // Number of parasites in first generation

t2 <- t23 * t2of3;

t3 <- t23 * (1 - t2of3);

for(i in 1:Nind) m1_log[i] <- M1_log_mu + M1_sd * m1_raw[i];

}

model {

vector[N_cens1_d20] log_pf_ml_cens1_d20_mu; // Log expected value of censored

// observations before first positives

// (dlimit = 20)

vector[N_cens1_d200] log_pf_ml_cens1_d200_mu; // (dlimit = 200)

vector[N_cens2_d20] log_pf_ml_cens2_d20_mu; // Log expected value of censored

// observations after first positives

// (dlimit = 20)

vector[N_cens2_d200] log_pf_ml_cens2_d200_mu; // (dlimit = 200)

vector[N_firstpos] log_pf_ml_firstpos_mu; // Log expected value of first positive

// observations

vector[N_cyc1plus] log_pf_ml_cyc1plus_mu; // Log expected value of positive

// observations after the first positive

// observation + known bloodsmear

vector[N_cyc1plus_TS_NA] log_pf_ml_cyc1plus_TS_NA_mu; // + unknown bloodsmear

vector[N_cens1_d20] int_cens1_d20; // Random intercepts for bloodsmear positivity

vector[N_cens1_d200] int_cens1_d200;

vector[N_cens2_d20] int_cens2_d20;

vector[N_cens2_d200] int_cens2_d200;

vector[N_firstpos] int_firstpos;

vector[N_cyc1plus] int_cyc1plus;

// Calculate expected log parasite concentrations per ml

// blood and random intercepts for bloodsmear positivity

for (i in 1:N_cens1_d20) {

log_pf_ml_cens1_d20_mu[i] <- log_Pf_ml_one_cycle(time_cens1_d20[i],

t1[id_cens1_d20[i]],

t2,

sigma_t1,

m1_log[id_cens1_d20[i]]);

int_cens1_d20[i] <- a + a_raw[id_cens1_d20[i]] * a_sd;

}

for (i in 1:N_cens1_d200) {

log_pf_ml_cens1_d200_mu[i] <- log_Pf_ml_one_cycle(time_cens1_d200[i],

t1[id_cens1_d200[i]],

t2,

sigma_t1,

m1_log[id_cens1_d200[i]]);

int_cens1_d200[i] <- a + a_raw[id_cens1_d200[i]] * a_sd;

}

for(i in 1:N_cens2_d20) {

log_pf_ml_cens2_d20_mu[i] <- log_Pf_ml(time_cens2_d20[i],

t1[id_cens2_d20[i]],

t2,

t3,

sigma_t1,

m1_log[id_cens2_d20[i]],

r[id_cens2_d20[i]],

Ncyc);

int_cens2_d20[i] <- a + a_raw[id_cens2_d20[i]] * a_sd;

}

for(i in 1:N_cens2_d200) {

log_pf_ml_cens2_d200_mu[i] <- log_Pf_ml(time_cens2_d200[i],

t1[id_cens2_d200[i]],

t2,

t3,

sigma_t1,

m1_log[id_cens2_d200[i]],

r[id_cens2_d200[i]],

Ncyc);

int_cens2_d200[i] <- a + a_raw[id_cens2_d200[i]] * a_sd;

}

for(i in 1:N_firstpos) {

log_pf_ml_firstpos_mu[i] <- log_Pf_ml_one_cycle(time_firstpos[i],

t1[id_firstpos[i]],

t2,

sigma_t1,

m1_log[id_firstpos[i]]);

int_firstpos[i] <- a + a_raw[id_firstpos[i]] * a_sd;

}

for(i in 1:N_cyc1plus) {

log_pf_ml_cyc1plus_mu[i] <- log_Pf_ml(time_cyc1plus[i],

t1[id_cyc1plus[i]],

t2,

t3,

sigma_t1,

m1_log[id_cyc1plus[i]],

r[id_cyc1plus[i]],

Ncyc);

int_cyc1plus[i] <- a + a_raw[id_cyc1plus[i]] * a_sd;

}

for(i in 1:N_cyc1plus_TS_NA) {

log_pf_ml_cyc1plus_TS_NA_mu[i] <- log_Pf_ml(time_cyc1plus_TS_NA[i],

t1[id_cyc1plus_TS_NA[i]],

t2,

t3,

sigma_t1,

m1_log[id_cyc1plus_TS_NA[i]],

r[id_cyc1plus_TS_NA[i]],

Ncyc);

}

// Increment the log-posterior

// Priors

sigma_t1 ~ normal(0, 0.2);

sigma ~ normal(0, 0.5);

t23 ~ lognormal(log(2), 1);

t2of3 ~ beta(65, 35);

T1_log_mu ~ normal(log(7), 0.05);

T1_sd ~ normal(0, 0.03);

t1 ~ lognormal(T1_log_mu, T1_sd);

M1_log_mu ~ normal(log(850), 1);

M1_sd ~ normal(0, 2);

m1_raw ~ normal(0, 1); // implies: m1_log ~ normal(M1_log_mu, M1_sd);

R_log_mu ~ normal(log(7.5), 1);

R_sd ~ normal(0, 0.4);

r ~ lognormal(R_log_mu, R_sd);

// Censored data likelihood (for computational efficiency, avoid

// integration and use estimated censored values, which are defined

// to be constrained below detection limit in the parameters block)

log_pf_ml_cens1_d20 ~ normal(log_pf_ml_cens1_d20_mu, sigma);

// implies: increment_log_prob(normal_cdf_log(dlimit20_log,

// log_pf_ml_cens1_d20_mu,

// sigma));

log_pf_ml_cens1_d200 ~ normal(log_pf_ml_cens1_d200_mu, sigma);

// implies: increment_log_prob(normal_cdf_log(dlimit200_log,

// log_pf_ml_cens1_d200_mu,

// sigma));

log_pf_ml_cens2_d20 ~ normal(log_pf_ml_cens2_d20_mu, sigma);

//implies: increment_log_prob(normal_cdf_log(dlimit20_log,

// log_pf_ml_cens2_d20_mu,

// sigma));

log_pf_ml_cens2_d200 ~ normal(log_pf_ml_cens2_d200_mu, sigma);

// implies: increment_log_prob(normal_cdf_log(dlimit200_log,

// log_pf_ml_cens2_d200_mu,

// sigma));

// Non-censored data likelihood

pf_ml_firstpos ~ lognormal(log_pf_ml_firstpos_mu, sigma);

pf_ml_cyc1plus ~ lognormal(log_pf_ml_cyc1plus_mu, sigma);

// Logistic regression of bloodsmear positivity vs. predicted log(Pf.ml)

a ~ normal(-5,5);

b ~ normal(0,5);

a_raw ~ normal(0,1); // implies random intercept ~ normal(a, a_sd);

a_sd ~ normal(0,2);

TS_cens1_d20 ~ bernoulli_logit(int_cens1_d20 + b * log_pf_ml_cens1_d20_mu);

TS_cens1_d200 ~ bernoulli_logit(int_cens1_d200 + b * log_pf_ml_cens1_d200_mu);

TS_cens2_d20 ~ bernoulli_logit(int_cens2_d20 + b * log_pf_ml_cens2_d20_mu);

TS_cens2_d200 ~ bernoulli_logit(int_cens2_d200 + b * log_pf_ml_cens2_d200_mu);

TS_firstpos ~ bernoulli_logit(int_firstpos + b * log_pf_ml_firstpos_mu);

TS_cyc1plus ~ bernoulli_logit(int_cyc1plus + b * log_pf_ml_cyc1plus_mu);

// no likelihood for cases with unknown bloodsmear results

}

generated quantities {

real T1_mu;

real M1_mu;

real m1[Nind];

real R_mu;

T1_mu <- exp(T1_log_mu);

M1_mu <- exp(M1_log_mu);

for(i in 1:Nind) m1[i] <- exp(m1_log[i]);

R_mu <- exp(R_log_mu);

}

# Stan code for log-linear sine model

functions {

}

data {

int<lower=0> Nind; // Number of individuals

int<lower=0> Nobs; // Number of uncensored observations

int<lower=0> id[Nobs]; // Individual to which observation pertains

vector<lower=0>[Nobs] time; // Time of observation

vector<lower=0>[Nobs] pf_ml; // Value of observation

int<lower=0> Nobs_200; // Number of observations under dlimit 200

int<lower=0> id_200[Nobs_200]; // Individual to which observation pertains

vector<lower=0>[Nobs_200] time_200; // Time of observation

vector<lower=0>[Nobs_200] pf_ml_200; // Value of observation

int<lower=0> Nobs_20; // Number of observations under dlimit 20

int<lower=0> id_20[Nobs_20]; // Individual to which observation pertains

vector<lower=0>[Nobs_20] time_20; // Time of observation

vector<lower=0>[Nobs_20] pf_ml_20; // Value of observation

real<lower=0> t23; // Assumed generation time

}

transformed data{

vector[2] mu_rfx; // mean vector of non-centered, correlated random effects for

// parasite multiplication rate and hypothetical load at time = 0

mu_rfx[1] <- 0;

mu_rfx[2] <- 0;

}

parameters {

// Parasite cycles, assuming Von Mises distribution for timing of cycle

real<lower=-2*pi(),upper=0> t_start_mu;

real<lower=0> t_start_k;

vector<lower=-2*pi(),upper=0>[Nind] t_start;

real<lower=0> ampl; // Amplitude of cycles

// Hypothetical number of parasites at time = 0

real M1_log_mu; // Average among individuals in population

real<lower=0> M1_sd; // Standard deviation among individuals in population

// Multiplication rate of parasites (per day, N.B. not per generation!!)

real<lower=0> R_log_mu; // Average among individuals in population

real<lower=0> R_sd; // Standard deviation among individuals in population

// Correlation between hypothetical number of parasites at time = 0 and multiplication rate

vector[2] rfx_raw[Nind];

real<lower=-1,upper=1> rho;

// Measurement error (log-scale)

real<lower=0> sigma;

// Censored observations

vector<upper=log(200)>[Nobs_200] log_pf_ml_200;

vector<upper=log(20)>[Nobs_20] log_pf_ml_20;

}

transformed parameters {

// Random effects for parasite concentrations

real m1_log[Nind];

real r_log[Nind];

for(i in 1:Nind) {

m1_log[i] <- M1_log_mu + M1_sd * rfx_raw[i, 1];

r_log[i] <- R_log_mu + R_sd * rfx_raw[i, 2];

}

}

model {

// Log expected values of blood parasite concentrations

vector[Nobs] log_pf_ml_mu;

vector[Nobs_200] log_pf_ml_mu_200;

vector[Nobs_20] log_pf_ml_mu_20;

matrix[2,2] tau_rfx;

for(i in 1:Nobs) {

log_pf_ml_mu[i] <- m1_log[id[i]] + time[i] * r_log[id[i]]

+ ampl * sin(2 * pi() * time[i] / t23 - t_start[id[i]]);

}

for(i in 1:Nobs_200) {

log_pf_ml_mu_200[i] <- m1_log[id[i]] + time_200[i] * r_log[id[i]]

+ ampl * sin(2 * pi() * time_200[i] / t23 - t_start[id[i]]);

}

for(i in 1:Nobs_20) {

log_pf_ml_mu_20[i] <- m1_log[id[i]] + time_20[i] * r_log[id[i]]

+ ampl * sin(2 * pi() * time_20[i] / t23 - t_start[id[i]]);

}

// Increment the log-posterior

// Priors

sigma ~ normal(0, 5);

t_start ~ von_mises(t_start_mu, t_start_k);

t_start_k ~ normal(0, 10);

// t_start_mu ~ uniform(0, 2*pi()); // already implied by bounds

// ampl \propto 1 | ampl > 0; // already implied by bounds

M1_log_mu ~ normal(-2, 5);

M1_sd ~ normal(0, 5);

R_log_mu ~ normal(log(7.5) / 2, 2);

R_sd ~ normal(0, 5);

tau_rfx[1, 1] <- 1.0;

tau_rfx[2, 2] <- 1.0;

tau_rfx[1, 2] <- rho;

tau_rfx[2, 1] <- rho;

rfx_raw ~ multi_normal(mu_rfx, tau_rfx); // implies: m1_log ~ normal(M1_log_mu, M1_sd);

// r_log ~ normal(R_log_mu, R_sd);

// corr(m1_log, r_log) = rho

// Likelihood (left-hand side is data)

pf_ml ~ lognormal(log_pf_ml_mu, sigma);

// Censored observations (left-hand side are parameters)

log_pf_ml_200 ~ normal(log_pf_ml_mu_200, sigma);

log_pf_ml_20 ~ normal(log_pf_ml_mu_20, sigma);

}

generated quantities {

real M1_mu;

real m1[Nind];

real R_mu;

real r[Nind];

M1_mu <- exp(M1_log_mu);

for(i in 1:Nind) m1[i] <- exp(m1_log[i]);

for(i in 1:Nind) r[i] <- exp(r_log[i]);

R_mu <- exp(R_log_mu);

}
