# Supplementary figures and images for: The Power of Malaria Vaccine Trials Using Controlled Human Malaria Infection
Source: PLoS Comput Biol. 2017 Jan 12;13(1):e1005255. doi: 10.1371/journal.pcbi.1005255 (PMC5230743; doi:10.1371/journal.pcbi.1005255)

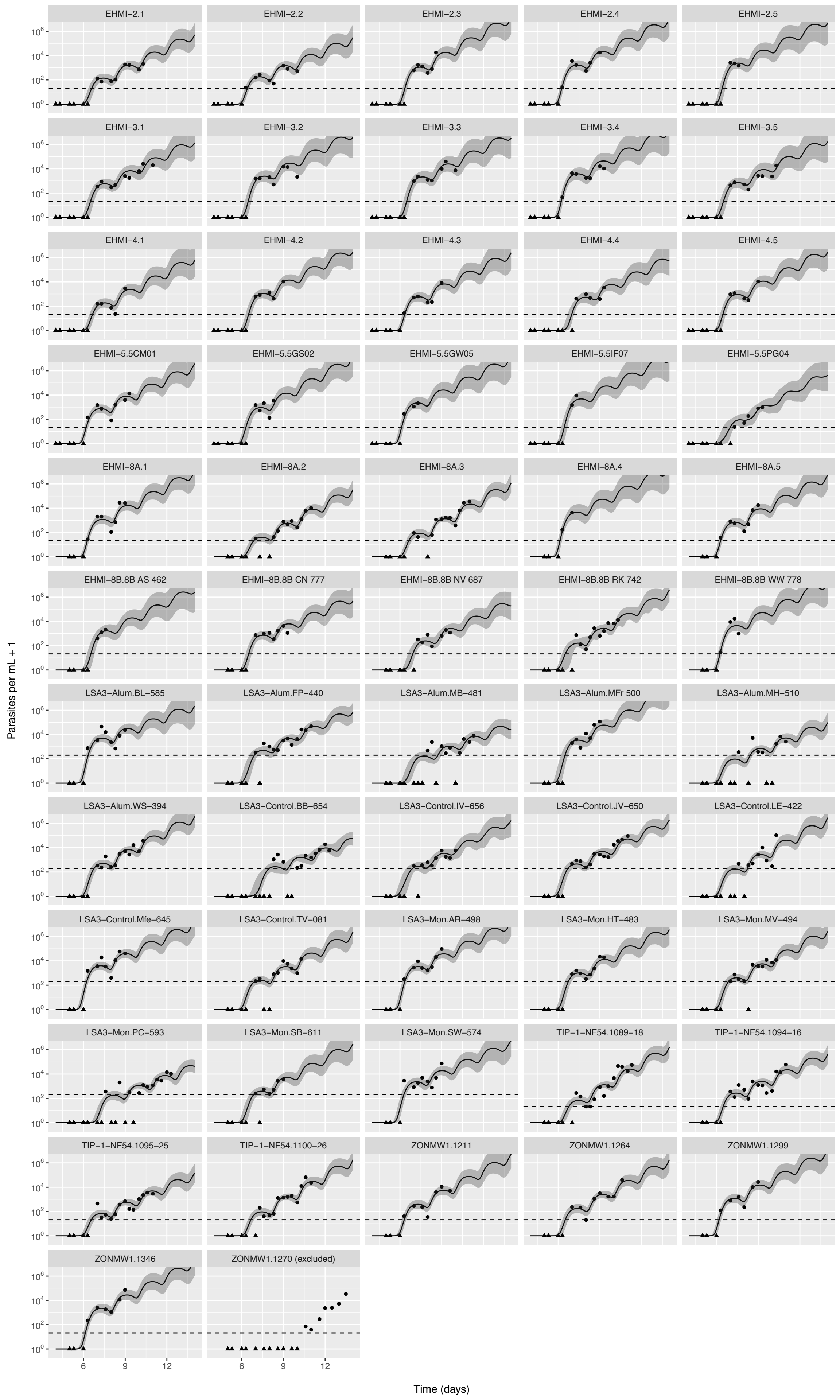

Supplement: S1 Fig — Solid lines represent the posterior mean; shaded bands represent the 2.5th and 97.5th percentiles of the predicted parasite concentrations, based on 8000 draws from the posterior distribution. Panel headers refer to unique identifiers for CHMI volunteers, which can also be found in the data (S1 File). (PDF) [file pcbi.1005255.s002.pdf]

Probability of positive blood smear (%)

1.00  
0.75  
0.50  
0.25  
0.00

$10^1$

$10^2$

$10^3$

$10^4$

$10^5$

Parasites per mL + 1

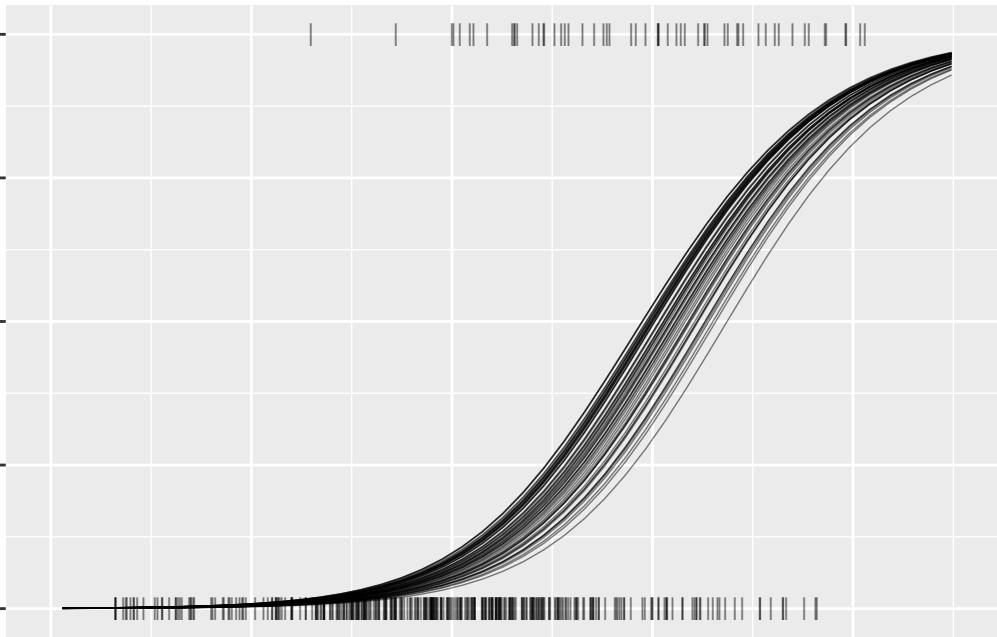

Supplement: S2 Fig — Vertical bars represent observed data on blood smear positivity (0/1) and parasite concentration. Note that the predicted probability of blood smear positivity is based on model-predicted parasite concentration; sigmoid lines represent predicted probabilities for different individuals and are each based on the mean of 8000 posterior draws. (PDF) [file pcbi.1005255.s003.pdf]

## Pre-erythrocytic vaccine trials

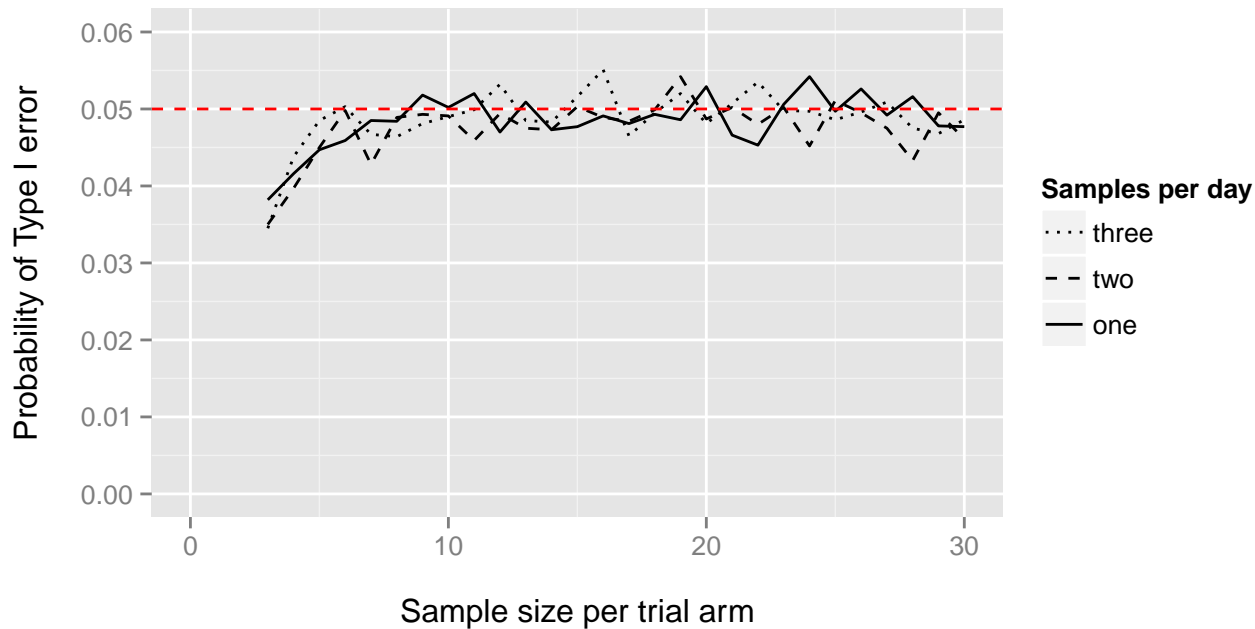

## Erythrocytic vaccine trials

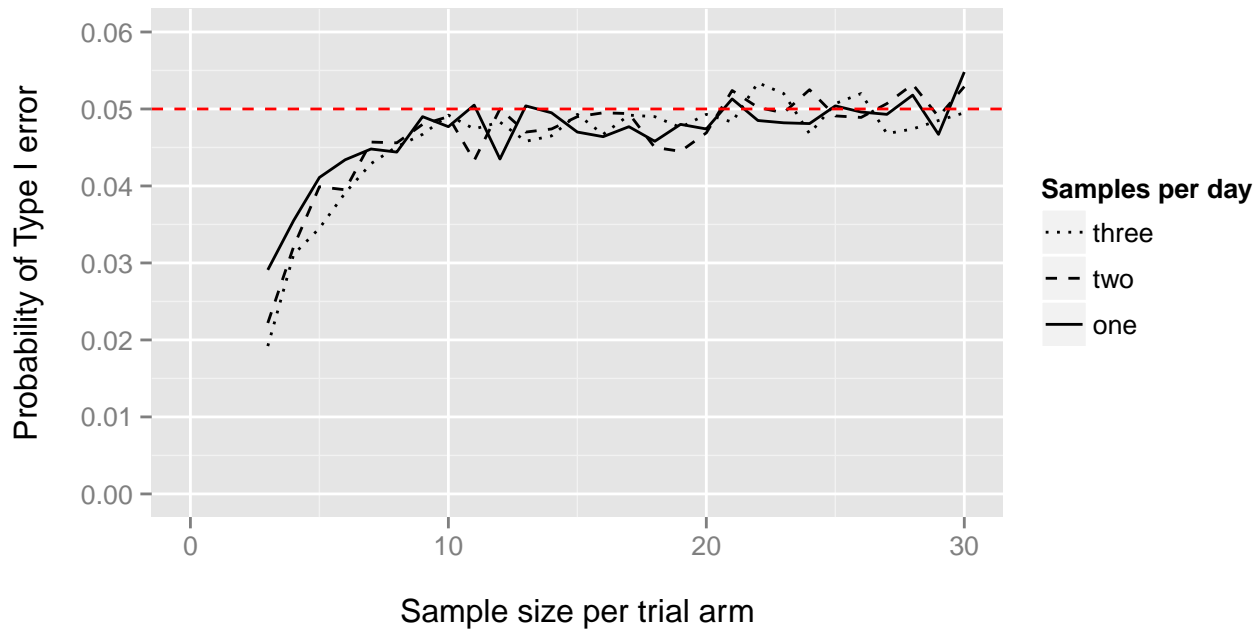

Supplement: S3 Fig — (PDF) [file pcbi.1005255.s004.pdf]

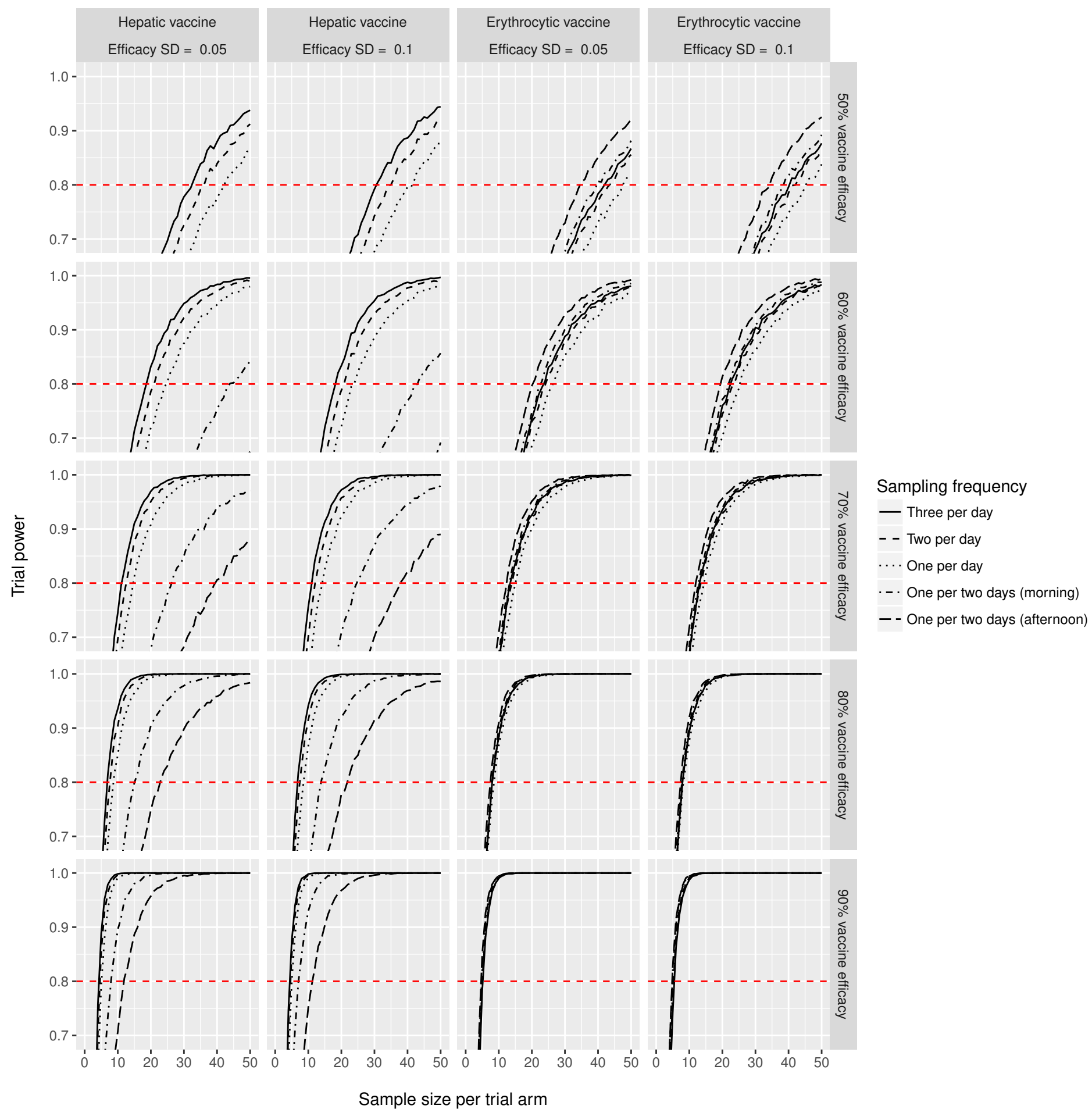

Supplement: S4 Fig — (PDF) [file pcbi.1005255.s005.pdf]

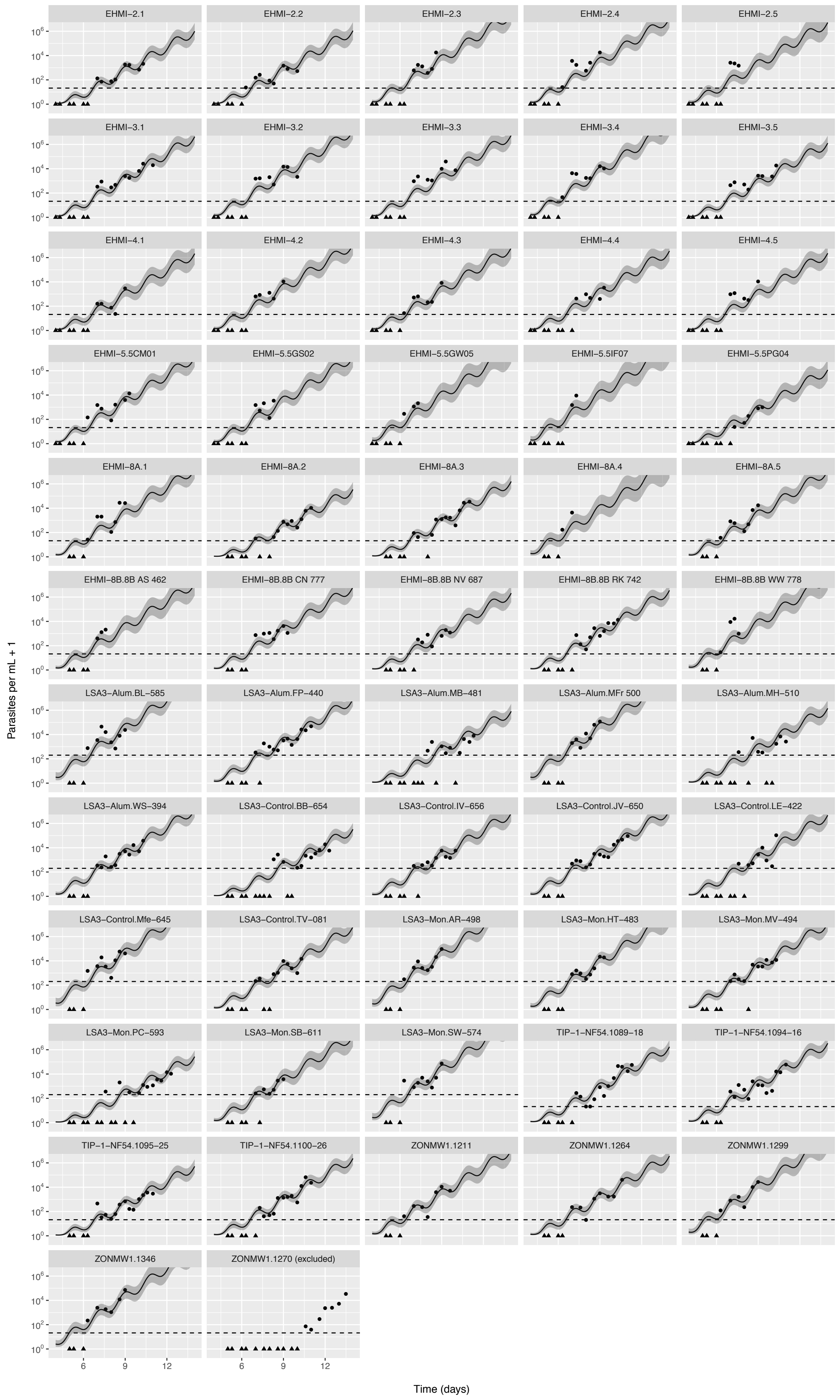

Supplement: S5 Fig — Solid lines represent the posterior mean; shaded bands represent the 2.5th and 97.5th percentiles of the predicted parasite concentrations, based on 8000 draws from the posterior distribution. Panel headers refer to unique identifiers for CHMI volunteers, which can also be found in the data (S1 File). (PDF) [file pcbi.1005255.s006.pdf]

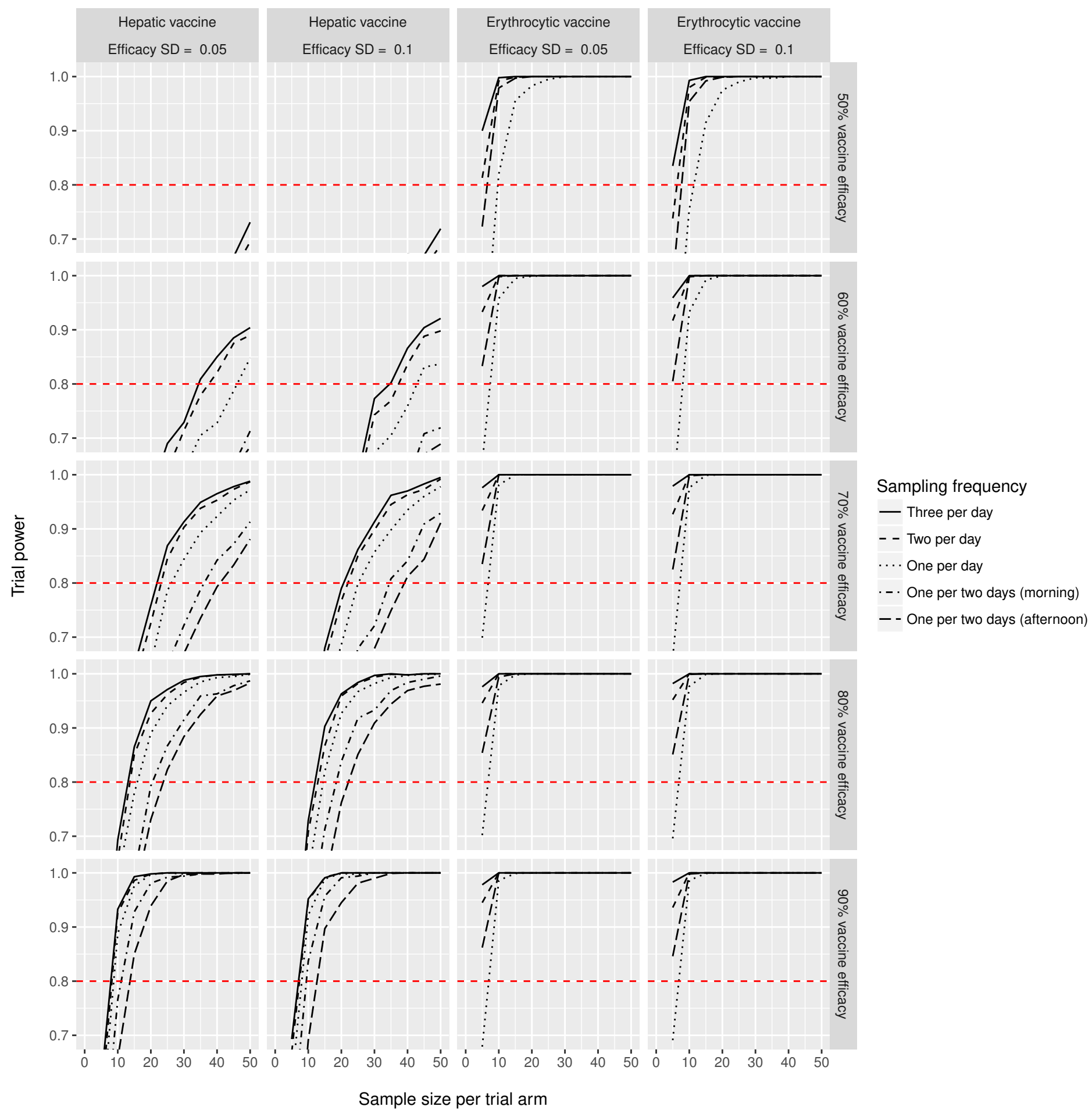

Supplement: S6 Fig — (PDF) [file pcbi.1005255.s007.pdf]
